# The UGT1A9*22 genotype identifies a high-risk group for irinotecan toxicity among gastric cancer patients

**DOI:** 10.5808/gi.22051

**Published:** 2022-09-30

**Authors:** Choong-kun Lee, Hong Jae Chon, Woo Sun Kwon, Hyo-Jeong Ban, Sang Cheol Kim, Hyunwook Kim, Hei-Cheul Jeung, Jimyung Chung, Sun Young Rha

**Affiliations:** 1 Division of Medical Oncology, Yonsei Cancer Center, Yonsei University College of Medicine, Seoul 03722, Korea; 2 Brain Korea 21 Plus Project for Medical Science, Yonsei University College of Medicine, Seoul 03722, Korea; 3 KM Data Division, Korea Institute of Oriental Medicine, Daejeon 34054, Korea; 4 Division of Healthcare and AI, Center for Precision Medicine, Korea National Institute of Health, Korea Centers for Disease Control and Prevention, Seoul 28159, Korea; 5 Department of Internal Medicine, Gangnam Severance Hospital, Yonsei University College of Medicine, Seoul 06273, Korea; 6 Yonsei University Graduate School, Yonsei University College of Medicine, Seoul 03722, Korea; 7 Songdang Institute for Cancer Research, Yonsei University College of Medicine, Seoul 03722, Korea

**Keywords:** DNA polymorphism, gastric neoplasms, irinotecan, toxicity, UGT1A9*22

## Abstract

Several studies have shown associations between irinotecan toxicity and *UGT1A* genetic variations in colorectal and lung cancer, but only limited data are available for gastric cancer patients. We evaluated the frequencies of *UGT1A* polymorphisms and their relationship with clinicopathologic parameters in 382 Korean gastric cancer patients. Polymorphisms of UGT1A1*6, UGT1A1*27, UGT1A1*28, UGT1A1*60, UGT1A7*2, UGT1A7*3, and UGT1A9*22 were genotyped by direct sequencing. In 98 patients treated with irinotecan-containing regimens, toxicity and response were compared according to the genotype. The UGT1A1*6 and UGT1A9*22 genotypes showed a higher prevalence in Korean gastric cancer patients, while the prevalence of the UG1A1*28 polymorphism was lower than in normal Koreans, as has been found in other studies of Asian populations. The incidence of severe diarrhea after irinotecan-containing treatment was more common in patients with the UGT1A1*6, UGT1A7*3 and UGT1A9*22 polymorphisms than in controls. The presence of the UGT1A1*6 allele also showed a significant association with grade III–IV neutropenia. Upon haplotype and diplotype analyses, almost every patient bearing the UGT1A1*6 or UGT1A7*3 variant also had the UGT1A9*22 polymorphism, and all severe manifestations of *UGT1A* polymorphism-associated toxicity were related to the UGT1A9*22 polymorphism. By genotyping UGT1A9*22 polymorphisms, we could identify high-risk gastric cancer patients receiving irinotecan-containing chemotherapy, who would experience severe toxicity. When treating high-risk patients with the UGT1A9*22 polymorphism, clinicians should closely monitor them for signs of toxicity such as severe diarrhea or neutropenia.

## Introduction

Irinotecan is commonly used alone or in combination with other medications to treat various cancers, including colorectal, gastric, and lung cancers. Genetic polymorphisms in genes involved in the metabolism of irinotecan, especially variations in the gene encoding UDP-glucuronosyl transferase 1A (*UGT1A*), are known to play significant roles in the outcomes of patients after treatment. Irinotecan and its active metabolite SN-38 (7-ethyl-10-hydroxy-camptothecin), which is approximately 100- to 1,000-fold more cytotoxic than the parent drug, are topoisomerase I inhibitors and cause cancer cell death as a result of DNA strand breaks induced via cleavable complexes [1]. Systemic SN-38 has been reported to cause neutropenia, whereas local intestinal SN-38 causes diarrhea [2]. Because neutropenia and diarrhea are the major toxicities associated with irinotecan chemotherapy, inherited differences in *UGT1A* polymorphisms affecting its activity may exert an important influence on the pharmacokinetics, toxicity, and pharmacologic effects of irinotecan. Some of these isoforms have been shown to have clinical significance for SN-38 glucuronidation and irinotecan-related toxicity; in particular, the low-activity *UGT1A1* isoform, UGT1A1*28, is strongly associated with irinotecan-induced neutropenia [3,4], especially in Western populations. Some studies have suggested that UGT1A1*6 or UGT1A1*27 might be more important among Asian cancer patients treated with irinotecan [5,6]. The –3279T>G (UGT1A1*60) and 211G>A (UGT1A1*6) polymorphisms have been suggested to be important with respect to UDP glucuronosyl transferase enzyme function [7]. In addition to UGT1A1, the extra-hepatic isoform UGT1A7 has been demonstrated to glucuronidate some phenolic compounds, carcinogens, and drugs. UGT1A7*3 was found to be a risk factor for oropharyngeal cancer, hepatocellular carcinoma, and colorectal cancer (CRC) [8], and a recent study showed that UGT1A7 plays a role in the glucuronidation of SN-38. Hepatic UGT1A9 has also been suggested to contribute to the metabolism of SN-38 [9], and one of the variants in its gene, ‒118(T)_10_/(T)_9_ (UGT1A9*22), is associated with a change in the enzyme phenotype [10,11].

Several studies have shown an association between toxicity and *UGT1A* genetic variations in CRC and lung cancer, but few studies have reported gastric cancer patients’ *UGT1A* polymorphisms and irinotecan toxicity [12,13]. Because gastric cancer is the second leading cause of cancer death worldwide, with an especially high incidence in Asia, it is important to identify *UGT1A* polymorphism candidates for predicting irinotecan toxicity among gastric patients [14]. Moreover, few studies have investigated isoforms other than *UGT1A1* and their relationships with irinotecan-induced toxicity. Since important candidate *UGT1A* polymorphisms, including UGT1A1*6, UGT1A1*27, UGT1A1*28, UGT1A1*60, *UGT1A7*, and UGT1A9*22, may play key roles in irinotecan metabolism, we evaluated the frequencies of *UGT1A* polymorphisms and their association with toxicity and other parameters in Korean gastric cancer patients. Furthermore, we also conducted a comparative analysis of toxicity according to the genotype among gastric cancer patients treated with irinotecan-containing regimens.

## Methods

### Subjects

Polymorphisms of *UGT1A* subtypes were investigated in 382 Korean patients with histologically confirmed gastric carcinoma. The patients’ clinical data were reviewed from the electronic medical records of Severance Hospital, Yonsei University College of Medicine, in accordance with the Declaration of Helsinki, after institutional review board approval. Among them, 98 patients with advanced gastric adenocarcinoma were enrolled in two prospective phase II studies and received irinotecan and cisplatin combination chemotherapy as second- or third-line therapy with palliative aims between April 2002 and August 2007. Forty-nine patients received a weekly regimen (50 mg/m^2^ irinotecan on days 1, 8, and 15, followed by 30 mg/m^2^ cisplatin on days 1, 8, 15, and a 1-week rest, every 4 weeks) and 49 patients received a biweekly regimen (70 mg/m^2^ irinotecan on days 1 and 15, and 80 mg/m^2^ cisplatin on day 1, every 4 weeks). The full treatment schedule, eligibility criteria, and dose modifications have been reported previously [15,16]. Toxicity was graded by the National Cancer Institute Common Toxicity Criteria version 3.0 for the most serious toxicity that a patient experienced during total therapy. Tumor response was evaluated according to the guidelines of the Response Evaluation Criteria in Solid Tumors Committee.

### Genomic DNA extraction and DNA genotyping

Genomic DNA was extracted from peripheral blood mononuclear cells (PBMCs) at the time of diagnosis. The PBMCs were isolated from whole blood using Ficoll-Paque (Pharmacia, Uppsala, Sweden), and genomic DNA was extracted with the LaboPass Blood kit (Genotein Biotech, Seoul, Korea) following the manufacturer’s instructions. The UGT1A1*28, UGT1A7*2, UGT1A7*3, and UGT1A9*22 polymorphisms were genotyped by direct sequencing, UGT1A1*27 by SNaPshot sequencing, and UGT1A1*6 and UGT1A1*60 using TaqMan probes.

Polymerase chain reaction (PCR) was performed according to the manufacturer’s instructions using a thermal cycler (GeneAmp 9700, Applied Biosystems, Foster City, CA, USA) with a final reaction volume of 10 µL containing 5 ng of genomic DNA, 0.5 U of Taq DNA polymerase (Qiagen, Hilden, Germany), 0.24 µM concentrations of each primer, and 0.2 mM dNTP. The sense and antisense primers are described in Supplementary Table 1. The cycling protocol began with a denaturation step of 94°C for 3 min, followed by 30 cycles of denaturation at 94°C for 30 s, annealing at the specific annealing melting temperature (60°C or 65°C) for 30 s, and 72°C for 1 min, with a final extension at 72°C for 10 min. The PCR products were purified by incubation with 0.15 U of shrimp alkaline phosphatase (Roche, Basel, Switzerland) and 0.75 U of exonuclease 1 (New England Biolabs, Ipswich, MA, USA) at 37°C for 45 min, followed by heat inactivation at 80°C for 15 min. The PCR products were sequenced using an ABI BigDye Terminator Cycle Sequencing Kit (Applied Biosystems) on an ABI 3100 DNA sequencer (Applied Biosystems).

SNaPshot analysis was performed using an Applied Biosystems SNaPshot Multiplex Kit. Extension reactions were performed in a thermal cycler and consisted of 35 cycles of denaturation at 95°C for 10 s and annealing/extension at 60°C for 40 s. The products were denatured at 95°C for 5 min and then separated using an ABI 3730xl DNA Analyzer (Applied Biosystems) with a 36-cm-long capillary and POP-7 polymer. The analysis was performed using GeneMapper 3.7 Software.

For the TaqMan Drug Metabolism Genotyping Assay, we performed genotyping using solution-phase hybridization reactions with 5′-nuclease and fluorescence detection (TaqMan SNP genotyping assay numbers C_559715_20 and C_1432134_10; Applied Biosystems) in a 7300 real-time PCR system (Applera, Norwalk, CT, USA). The 25 µL reactions contained 20 ng of genomic DNA, 1× TaqMan Universal Master Mix, 900 nM concentrations of each primer, and 200 nM VIC-labeled and FAM-labeled probes (Applera). The amplification conditions were 95°C for 10 min, then 40 cycles of 92°C for 15 s, and 60°C for 1 min.

### Data analyses

Genotypes for various polymorphisms were assessed for deviation from Hardy-Weinberg equilibrium using SNP Analyzer software (http://www.istech21.com/bionics/consulting_6.htm). We analyzed linkage disequilibrium (LD) using the PLINK program (http://pngu.mgh.harvard.edu/~purcell/plink), which gave the following LD statistics: r-square, D′, haplotype, and estimated haplotype frequencies, in-phase. The LD statistics presented in the PLINK results are based on haplotype frequencies estimated via an expectation-maxmization algorithm. Diplotypes were assigned to each individual from their haplotypes.

The genotype frequencies of *UGT1A* polymorphisms in gastric cancer patients were compared with previously reported data. The associations between *UGT1A* genotypes and clinical parameters, toxicity, and response were compared using the Pearson chi-square test or Fisher exact test, using SPSS version 21.0 (IBM Corp., Armonk, NY, USA). A p-value < 0.05 was considered to indicate statistical significance.

## Results

### Baseline characteristics of 382 patients

*UGT1A* polymorphism subtypes were determined in 382 gastric cancer patients. The study sample included 247 men and 135 women with a median age of 57 years (range, 27 to 86 years), and included patients with stage I (35.1%), II (9.2%), III (19.1%), and IV (36.6%) cancers. Poorly differentiated adenocarcinoma was the most common histological type (128 patients, 33.5%). No patients were previously diagnosed with Gilbert syndrome.

### *UGT1A* genotypes and frequencies

UGT1A1*6, UGT1A1*27, UGT1A1*28, UGT1A1*60, and UGT1A7*3 variants (N129K, R131R/K, R131Q/K, and W208R), and UGT1A9*22 were genotyped in 382 patients. Their frequencies are listed in Table 1, which shows homozygous wild-type (w/w), heterozygous (w/m), and homozygous mutant (m/m) types. All variants were in Hardy-Weinberg equilibrium (p > 0.05).

The *UGT1A* polymorphism allele frequencies of gastric cancer patients in our study were compared with those in previously reported data from normal Koreans [17,18] and Koreans with non‒small cell lung cancer (NSCLC) [11] or CRC [19]. The *UGT1A* polymorphism allele frequencies in Asians and normal Europeans [10, 20] were also compared with those of Japanese patients with various cancers [21] and with Western patients with metastatic CRC [22], as shown in Table 2. The frequency of the UGT1A*6 allele was significantly higher in Korean cancer patients, especially for gastric cancer patients (37.4%, p = 0.015) and NSCLC patients (39.5%, p = 0.020), than in normal Koreans. Data from Asian patients showed that cancer patients had a similar frequency of the UGT1A1*6 allele compared to normal individuals (16.7% vs. 13%), but those prevalence rates were lower than observed in Korean cancer patients. Unlike the Asian data, Western individuals had no UGT1A1*6 polymorphisms. The prevalence of the UGT1A1*28 polymorphism was lower in Korean gastric cancer patients (23.8%, p = 0.775), similar to other Korean and Asian populations (11.0%‒25%), and different from studies of Western populations (>30.0%). The incidence of the UGT1A1*60 polymorphism was significantly higher in Korean gastric cancer patients (50.8%, p < 0.001) than in normal Koreans (26.0%). Other cancer types also showed a higher prevalence of UGT1A1*60 (NSCLC, 43.2%, p = 0.004; CRC, 55.0%, p = 0.001) than in normal groups, which were different from other Asian populations (28.0%–34.0%, p = 0.106). In contrast, the UGT1A7*3 genotype did not show any significant difference between normal Koreans and cancer patients. The UGT1A9*22 polymorphism showed similarly higher frequencies across Korean normal and gastric cancer patients (66.0%–64.1%, p = 0.876). Japanese and Western cancer patients also showed significantly high frequencies of the UGT1A9*22 allele (65.3%, p = 0.002 and 56.8%, p = 0.032) compared to normal individuals (53.0% and 39.0%).

### Linkage disequilibrium and haplotype analysis

**Fig. 1.**
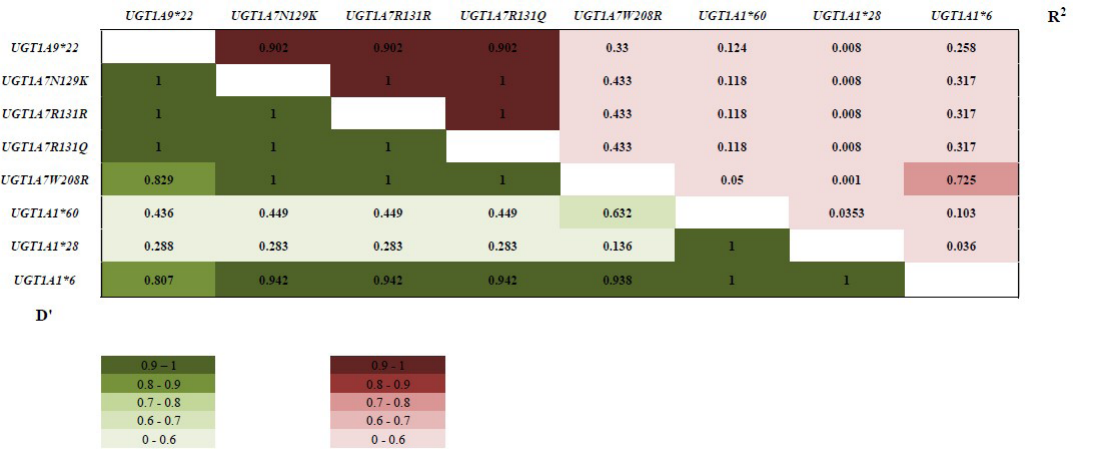
Linkage disequilibrium analysis for *UGT1A* polymorphisms. Linkage disequilibrium analysis was performed using the detected single-nucleotide polymorphisms. In particular, a close linkage was seen between UGT1A9*22 and *UGT1A7* variants (D’ = 1, R^2^ = 0.902). UGT1A1*6 was also highly linked with other *UGT1A* polymorphisms (D’ = 0.807–1).

Since LD (the non-random association of alleles at two loci) shows considerable heterogeneity across the human genome, we performed LD analyses using the detected SNPs (Fig. 1). In particular, close linkage was seen between UGT1A9*22 and UGT1A7 variants (D′ value = 1; r^2^ value = 0.902). UGT1A1*6 was also highly linked with other *UGT1A* polymorphisms (D′ values of 0.807–1).

Haplotype and diplotype analyses are the best ways to understand LD patterns in the human genome, reflecting the combined effects at multiple loci throughout the evolutionary process. Haplotype analysis was performed with five *UGT1A* polymorphisms, resulting in 15 total reconstructed haplotypes. The haplotype frequencies of the total 382 patients, 98 of whom received chemotherapy containing irinotecan, are shown in Supplementary Table 2. The three most common haplotypes (I, II, and III) accounted for over 80% of all haplotypes.

A total of 41 diplotypes were found, and only those with frequencies over 1% among the 382 patients are shown in Supplementary Table 3. The four most common diplotypes were I/I (22.77%), I/II (17.80%), I/III (15.18%), and I/IV (8.64%). Similar results were observed among the 98 patients who received irinotecan-containing chemotherapy.

### Characteristics of patients who were treated with irinotecan-based chemotherapy

Ninety-eight patients received combination chemotherapy of irinotecan and cisplatin with palliative aims, and their baseline characteristics are shown in Table 3. Among these 98 patients, 62 (63.3%) were men and the overall median age was 57 years (range, 27 to 76 years). Forty-nine patients received chemotherapy as a weekly regimen with a relative dose intensity (RDI) of 85.5% (standard deviation [SD] = 25.1%; range, 33.3% to 116.7%), whereas 49 patients received chemotherapy on a biweekly regimen with a RDI of 80.3% (SD = 19.3%; range, 43.8% to 132.9%). Approximately 14.3% (14/98) of the patients experienced grade III–IV diarrhea, and 28.6% (28/98) of the patients experienced grade III–IV neutropenia. Interestingly, the weekly regimen group and biweekly regimen group showed distinct toxicity profiles. The biweekly regimen group seemed to experience more toxicity than the weekly group, with a higher incidence of grade III–IV diarrhea (11 [22.4%] vs. 3 [6.1%] patients) and grade III–IV neutropenia (21 [42.9%] vs. 7 [14.3%] patients).

We also analyzed the associations of each of the *UGT1A* genotypes with clinical parameters, including age, sex, histology, stage, and baseline total bilirubin. The genotypes did not show any statistically significant correlation with any of these clinical parameters (data not shown).

### Association of *UGT1A* genotypes with toxicity and tumor response to irinotecan-containing treatment

The associations between each genotype and toxicity for the 98 patients who received palliative irinotecan-based chemotherapy are shown in Table 4. In this analysis, additional genotyping for the UGT1A1*27 polymorphism was also done, because previous studies have reported that Japanese cancer patients heterozygous for UGT1A1*27 experienced severe diarrhea or leukopenia [6]. Patients with the UGT1A1*6 polymorphism showed a significantly higher incidence of grade III–IV diarrhea (9/34 patients [26.47%], p = 0.012) and grade III–IV neutropenia (14/34 patients [41.18%], p = 0.044) after the irinotecan-containing regimen. Patients with the UGT1A7*3 polymorphism and the UGT1A9*22 polymorphism also showed higher incidence rates of grade III–IV diarrhea (10/41 patients (24.39%), p = 0.041, and 13/68 patients (19.12%), p = 0.033, respectively). However, the other polymorphisms did not show any significant associations with grade III–IV diarrhea or grade III–IV neutropenia.

To determine whether the different regimens, with different irinotecan doses and schedules, affected the pharmacogenetic associations with *UGT1A*, we compared the genotypes of the two treatment arms with respect to toxicity data. Interestingly, even though the incidence of toxicity was higher among patients receiving the biweekly regimen, the weekly regimen seemed to contribute more to the significance of the *UGT1A* polymorphisms’ relationship to higher toxicity. The presence of the UGT1A1*6 allele and UGT1A7*3 allele showed significant associations with grade III–IV diarrhea among patients who received the weekly regimen (p = 0.02, and p = 0.049), but not among those who received the biweekly regimen. In patients who experienced grade III–IV neutropenia, the UGT1A1*6, UGT1A7*3 and UGT1A9*22 polymorphisms showed significant associations only among those who received the weekly regimen (p = 0.015, p = 0.042, and p = 0.024, respectively).

**Fig. 2.**
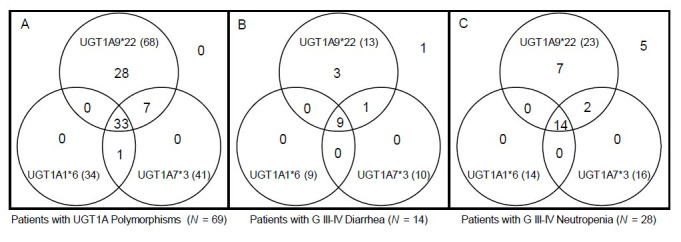
Venn diagram showing the distribution of *UGT1A* polymorphisms, patients with severe diarrhea, and patients with severe neutropenia (total = 98). A Venn diagram shows distribution of *UGT1A* polymorphism patients, and their relationship with severe diarrhea and neutropenia. Almost every patient had the UGT1A9*22 polymorphism with or without the UGT1A1*6 or UGT1A7*3 polymorphism (A). Among patients with any *UGT1A* polymorphism, those who suffered severe toxicity all had the UGT1A9*22 polymorphism (B, C).

For 98 patients, we constructed diplotypes based on haplotype analyses only with the significantly toxicity-related *UGT1A* polymorphisms: UGT1A1*6, UGT1A7*3 and UGT1A9*22. In total, 12 diplotypes were constructed, and the associations between each diplotype and grade III–IV diarrhea or neutropenia are shown in Table 5. Interestingly, after excluding 29 patients with wild-type diplotypes, almost every patient (68 out of 69 patients [98.6%]) had the UGT1A9*22 polymorphism with or without the UGT1A1*6 or UGT1A7*3 polymorphism. Among patients who had any of the UGT1A1*6, UGT1A7*3 or UGT1A9*22 variants, 13 suffered grade III–IV neutropenia and 23 suffered grade III–IV diarrhea. All patients who suffered severe toxicity associated with significant *UGT1A* polymorphisms had the UGT1A9*22 polymorphism, as shown in a Venn diagram (Fig. 2).

Each haplotype was also assessed for its associations with clinical parameters, toxicity, and tumor response, but none of the haplotypes showed any statistically significant correlations (data not shown).

The associations of *UGT1A* genotypes with treatment response were also evaluated (Supplementary Table 4). Out of 91 assessable patients, 16 patients (17.6%) with complete or partial responses were considered responders. There were no associations between any *UGT1A* polymorphism, including haplotypes or diplotypes, and treatment response to irinotecan-containing chemotherapy. In addition, neither treatment regimen–based group showed any significant associations between genotypes and responses.

## Discussion

Many studies have shown that, besides tumor-specific genes, individual genetic variations can also act as important predictors of tumor response and toxicity when treating patients with antitumor agents. For instance, with irinotecan treatment, up to 50% of Western patients with the UGT1A1*28 allele suffered from severe neutropenia [3,4], and these findings led to the recommendation to consider a reduced initial dose for patients known to be homozygous for the UGT1A1*28 allele. As such, it is very important to understand ethnic differences between genotypes for the development of predictive biomarkers.

In this study, we analyzed the frequencies of important subtypes of *UGT1A1, UGT1A7*, and *UGT1A9* in 382 Korean gastric cancer patients. As the Korean demographic profile shows relatively high ethnic homogeneity, we confirmed that the prevalence of *UGT1A* polymorphisms in Korean gastric cancer patients shows unique patterns compared with Western and other Asian populations. Korean cancer patients appeared to have similar frequency patterns of the *UGT1A* genotype irrespectively of cancer type and appeared to have a higher prevalence of UGT1A1*6 and UGT1A1*60 than normal Koreans. These results might be due to the large number of cases we investigated compared with previously reported data. The elevated prevalence of the UGT1A1*6 allele may be a common finding in Asians [10]. The UGT1A9*22 polymorphism has been frequently detected worldwide, especially among Korean gastric cancer patients. Upon haplotype analyses, the haplotype and diplotype distributions were similar to those of previously reported Korean studies [11], but differed from those in Caucasians or even other Asians [10]. These discrepancies in *UGT1A* polymorphism patterns between Koreans and Westerners or other Asians indicate that a personalized approach is needed for each patient, considering each individual’s genetic information in terms of irinotecan metabolism.

LD analyses showed that UGT1A1*6 exhibited a close linkage with *UGT1A7* variants, especially *UGT1A7W208R*, which has a mutation in UGT1A7*3 (R^2^ = 0.725, D′ = 0.938). The *UGT1A7* variants were also closely linked with UGT1A9*22 (R^2^ = 0.33–0.902, D′ = 0.829–1.0). These LD analyses might explain why patients with the UGT1A7*3 and UGT1A9*22 alleles also experienced more severe diarrhea when treated with irinotecan, similar to patients with the UGT1A1*6 allele. Previous LD analyses in Japanese cancer patients yielded similar results to those of our study [23]. Han et al. [11] reported a genotype-pharmacokinetics association analysis among irinotecan-treated Korean NSCLC patients, and the UGT1A1*6/*6, UGT1A7*3/*3, and *UGT1A9-118(T)9/9* genotypes were associated with significantly lower area under the time-concentration curve ratios of SN-38G to SN-38. This Korean irinotecan pharmacokinetics study might also explain our finding that UGT1A1*6, UGT1A7*3, and UGT1A9*22 were associated with severe diarrhea or neutropenia.

In agreement with a previous Korean study among NSCLC patients [11], our study showed that the UGT1A1*28 polymorphism was not related to the toxicity of irinotecan. This differs from a previous Japanese study [6], which reported a 3.5-fold higher frequency of the UGT1A1*28 allele in patients exhibiting toxicity than in patients without this complication. Previous Chinese studies also confirmed that, among esophageal cancer patients, both the UGT1A1*6 and UGT1A1*28 variants were related to severe neutropenia [12]. However, among Chinese gastric cancer patients treated with irinotecan, only the UGT1A1*6 variant was related to severe neutropenia. It is not clear whether the close association between irinotecan toxicity and the UGT1A1*6 polymorphism alone, but not the UGT1A1*28 polymorphism, is limited to Korean cancer patients or gastric cancer patients.

Although genotyping every patient who receives irinotecan-based chemotherapy is not currently recommended, there is no doubt that instituting such a practice would yield significant benefits. To do this, it would be important to know which polymorphisms we should genotype in order to predict who will suffer from irinotecan-related toxicity. The UGT1A1*6, UGT1A7*3 and UGT1A9*22 genotypes were shown to be related to severe toxicity concordant with a Japanese study of FOLFIRI-treated colorectal cancer patients [24]. When we constructed diplotypes based only on these three important *UGT1A* polymorphisms, we interestingly found that UGT1A9*22 was assigned to almost every diplotype if the patient had any one of those three *UGT1A* polymorphisms. This suggests that, by genotyping UGT1A9*22, we might expect to find any individuals with one of the *UGT1A* polymorphisms that are significantly related to irinotecan toxicity. To our knowledge, no previous study has shown this relationship or the importance of UGT1A9*22. Only one study was previously reported regarding the associations of *UGT1A* polymorphisms with irinotecan toxicity among gastric cancer patients [12]. However, only 42 patients were analyzed, with limitations such as heterogeneous chemotherapy regimens that were used, and the UGT1A9*22 polymorphism was not genotyped among patients in that previous study. Previously reported Asian data suggested that the UGT1A1*6 polymorphism was associated with severe irinotecan-related toxicity [5,11-13,24], which was never observed in Western populations. A more interesting finding is that patients who suffered severe forms of toxicity that have previously been known to be associated with any of the significant *UGT1A* polymorphisms all had the UGT1A9*22 polymorphism, which confers more importance to the UGT1A9*22 polymorphism as a predictive marker for gastric cancer patients at high risk for irinotecan toxicity. Since a recent report concluded that *UGT1A1* polymorphisms are more important in liver SN-38 glucuronidation than *UGT1A9* [25], our conclusion highlighting the importance of UGT1A9*22 may not be because of its function but because of its close linkage with UGT1A1*6 and UGT1A7*3. Nonetheless, in view of the ability to predict toxicity, UGT1A9*22 will act as a good surrogate marker. Although only about 20% to 30% of patients who had the UGT1A9*22 polymorphism suffered severe toxicity, by classifying these patients as a high-risk group for irinotecan toxicity, clinicians could follow these patients more closely for severe diarrhea or neutropenia. Screening for UGT1A1*6 indeed identifies some high-risk patients, but it still cannot account for the majority; whereas screening for UGT1A9*22 will reveal a higher proportion of high-risk patients.

Choosing the optimal drug dose levels is important in chemotherapy. Stewart et al. [26] showed that the *UGT1A1* promoter genotype had no correlation with severe toxicity when patients received low-dose (15–75 mg/m^2^) irinotecan. It is also important to consider the regimen and treatment plan, especially when combining irinotecan with other agents. Table 4 clearly shows that the biweekly regimen was associated with a higher incidence of severe diarrhea or neutropenia. There was no significant difference in the delivered dose of irinotecan (weekly regimen, mean = 32.1 mg/m^2^/wk [range, 12.5 to 84.7]; biweekly regimen, mean = 32.1 mg/m^2^/wk [range, 17.5 to 53.1]), and relative dose intensity (weekly regimen, 85.5%; biweekly regimen, 86.9%). Combining drugs might also play an important role, because platinum anti-neoplastic agents also induce gastrointestinal toxicity and myelo-suppression. One more factor to consider is whether a reduced dose of irinotecan has efficacy in patients with certain *UGT1A* polymorphisms (for example, UGT1A1*28 for Europeans or UGT1A1*6 and UGT1A9*22 for Koreans), or whether they should receive an alternative drug. A well-designed large prospective study might be needed to determine the optimal dose and regimen based on pharmacogenetics.

In summary, our study shows a unique *UGT1A* genotype pattern in Korean gastric cancer patients and suggests that the presence of the UGT1A1*6, UGT1A7*3 and UGT1A9*22 polymorphisms in gastric cancer patients receiving irinotecan-containing treatment was significantly associated with severe toxicity. Of particular note, using haplotype and diplotype analysis, we suggest that only by genotyping UGT1A9*22 polymorphisms will clinicians be able to identify high-risk patients who might suffer severe forms of toxicity significantly related to *UGT1A* polymorphisms, especially among gastric cancer patients receiving irinotecan-containing chemotherapy. Once these patients are recognized as being a high-risk group for irinotecan toxicity, we can closely monitor them for severe diarrhea or neutropenia and provide appropriate management. Even though we are in a targeted-agent era, conventional cytotoxic agents still play major roles in treating cancer patients. Our study results indicate that a pharmacogenetic-based approach could lead to effective personalized anticancer treatment for advanced gastric cancer patients, especially when they are treated with an irinotecan-containing regimen.

## Figures and Tables

**Table 1.** Frequencies of *UGT1A* polymorphisms among 382 gastric cancer patients

Gene	Polymorphism	No. of subjects (%)			HWE p-value
		‒/‒	‒/+	+/+	
*UGT1A1*	211G>A (*6)	239 (62.6)	133 (34.8)	10 (2.6)	0.09
	686C>A (*27)^a^	132 (97.8)	3 (2.2)	0	0.77
	‒53(TA)6>7 (*28)	291 (76.2)	85 (22.3)	6 (1.6)	0.94
	‒3279T>G (*60)	188 (49.2)	165 (43.2)	29 (7.6)	0.38
*UGT1A7*	129N>K	126 (33.0)	197 (51.6)	59 (15.4)	0.21
	131R>R/K	126 (33.0)	197 (51.6)	59 (15.4)	0.21
	131R>Q/K	126 (33.0)	197 (51.6)	59 (15.4)	0.21
	208W>R	222 (58.1)	142 (37.2)	18 (4.7)	0.43
*UGT1A9*	‒118(T)_10_/(T)_9 _(*22)	137 (35.9)	194 (50.8)	51 (13.4)	0.17

*UGT1A*, uridine diphosphate-glucuronosyl transferase 1A; HWE, Hardy-Weinberg equilibrium.

a Only 135 patients' blood samples were available for genotyping for the UGT1A1*27 polymorphism.

**Table 2.** *UGT1A* allele frequencies of normal and various cancer patients among Koreans, Asians, and Westerners

	Koreans				Other Asians (Japanese)		Westerners	
	NL^a^	GC^b^	NSCLC^c^	CRC^d^	NL^e^	Cancer^f^	NL^e^	CRC^g^
No.	50	382	81	20	150	177	132	66
UGT1A1*6 (%)	20.0	37.4	39.5	25.0	13.0	16.7	0.0	-
p-value	-	0.015	0.02	0.645	-	0.226	-	-
UGT1A1*28 (%)	22.0	23.8	14.8	25.0	11.0	13.8	34.0	30.3
p-value	-	0.775	0.294	0.787	-	0.288	-	0.175
UGT1A1*60 (%)	26.0	50.8	43.2	55.0	34.0	28	44.0	-
p-value	-	<0.001	0.004	0.001		0.106	-	-
UGT1A7*3 (%)	36.0	41.9	44.4	-	26.0	22.3	36.0	31.1
p-value	-	0.427	0.34	-	-	0.357	-	0.322
UGT1A9*22 (%)	66.0	64.1	41.4	-	53.0	65.3	39.0	56.8
p-value	--	0.876	0.06	-	-	0.002	-	0.032

*UGT1A*, uridine diphosphate-glucuronosyl transferase 1A; NL, normal; GC, gastric cancer patients; NSCLC, non‒small cell lung cancer; CRC, colorectal cancer.

a Normal Korean data were from Yea et al. [17], except for data on *UGT1A*1*60 from Kim et al. [18] (n = 218).

b Korean gastric cancer data were from our study.

c Korean NSCLC data were from Han et al. [11].

d Korean CRC data were from Oh et al. [19].

e Normal Asian and Western data were from Innocenti et al. [10] except for data on *UGT1A*7*3 from Huang et al. [20] (n = 103).

f Japanese various cancer data were from Saito et al. [21].

g Western CRC data were from Carlini et al. [22].

**Table 3.** Baseline characteristics of patients treated with palliative chemotherapy containing irinotecan

Total patients entered	Total (n = 98)	Weekly regimen (n = 49)^a^	Biweekly regimen (n = 49)^b^
Sex			
Male	62 (63.3)	31 (63.3)	31 (63.3)
Female	36 (36.7)	18 (36.7)	18 (36.7)
Age at diagnosis (y)	57 (27-76)	57 (31-76)	56 (27-73)
Performance status (ECOG)			
0	7 (7.1)	0	7 (14.3)
1	71 (72.4)	38 (77.6)	33 (67.3)
2	17 (17.3)	9 (18.4)	8 (16.3)
3	3 (3.1)	2 (4.1)	1 (2.0)
Operation type			
No operation	26 (26.5)	16 (32.7)	10 (20.4)
Total gastrectomy	33 (33.7)	14 (28.6)	19 (38.8)
Subtotal gastrectomy	32 (32.7)	13 (26.5)	19 (38.8)
Open and closure	7 (7.1 )	6 (12.2)	1 (2.0)
Histology type			
Adenocarcinoma, well differentiated	2 (2.0 )	1 (2.0)	1 (2.0)
Adenocarcinoma, moderately differentiated	19 (19.4 )	9 (18.4)	10 (20.4)
Adenocarcinoma, poorly differentiated	55 (56.1)	27 (55.1)	28 (57.1)
Signet ring cell	18 (18.4)	9 (18.4)	9 (18.4)
Mucinous carcinoma	2 (2.0)	1 (2.0)	1 (2.0 )
Others	1 (1.0)	1 (2.0)	0
N/E	1 (1.0)	1 (2.0)	0
Diarrhea			
Grade 0, I, II	84 (85.7)	46 (93.9)	38 (77.6)
Grade III-IV	14 (14.3)	3 (6.1)	11 (22.4)
Neutropenia			
Grade 0, I, II	70 (71.4)	42 (85.7)	28 (57.1)
Grade III‒IV	28 (28.6)	7 (14.3)	21 (42.9)
Treatment response			
Complete response	3 (3.1)	3 (6.5)	0
Partial response	13 (13.3)	8 (17.4)	5 (11.1)
Stable disease	33 (33.7)	18 (39.1)	15 (33.3)
Progressive disease	42 (42.9)	17 (37.0)	25 (55.6)
N/E	7 (7.1)	3 (6.5)	4 (8.2)

Values are presented as number (%) or median (range). ECOG, Eastern Cooperative Oncology Group; NE, not evaluable.

a Weekly regimen: irinotecan (50 mg/m^2^) + cisplatin (30 mg/m^2^) (weekly for 3 weeks, with a 1-week rest).

b Biweekly regimen: irinotecan (70 mg/m^2^) every 2 weeks + cisplatin (80 mg/m^2^) every 4 weeks.

**Table 4.** Association of *UGT1A* genotypes with chemotherapy toxicity in 98 patients treated with Irinotecan-containing chemotherapy

		Total (n = 98)				GIII-IV diarrhea				GIII-IV neutropenia			
		GIII-IV diarrhea		GIII-IV neutropenia		Weekly regimen (n = 49)		Biweekly regimen (n = 49)		Weekly regimen		Biweekly regimen (n = 49)	
		No. (%)	p-value	No. (%)	p-value	No. (%)	p-value	No. (%)	p-value	No. (%)	p-value	No. (%)	p-value
UGT1A1*6			0.012		0.044		0.02		0.293		0.015		0.801
	w(-/-)	5/64 (7.8)		14/64 (21.9)		0/35 (0.0)		5/29 (17.2)		2/35 (5.7)		12/29 (41.4)	
	m(-/+, +/+)	9/34 (26.5)		14/34 (41.2)		3/14 (21.4)		6/20 (30.0)		5/14 (35.7)		9/20 (45.0)	
UGT1A1*27 ^a^			0.253		0.354		0.88		0.484		0.732		0.452
	w(-/-)	13/92 (14.1)		27/92 (29.4)		3/47 (6.4)		10/45 (22.2)		7/47 (14.9)		20/45 (44.4)	
	m(-/+, +/+)	1/2 (50.0)		1/2 (50.0)		0/0 (0.0)		1/2 (50.0)		0/0 (0.0)		1/2 (50.0)	
UGT1A1*28			0.46		0.772		0.612		0.386		0.895		0.779
	w(6/6)	11/72 (15.3)		20/72 (27.8)		2/36 (5.6)		9/36 (25.0)		5/36 (13.9)		15/36 (41.7)	
	m(6/7, 7/7)	3/26 (11.5)		8/26 (30.8)		1/13 (7.7)		2/13 (15.4)		2/13 (15.4)		6/13 (46.2)	
UGT1A1*60			0.741		0.949		0.547		0.465		0.571		0.74
	w(-/-)	7/45 (15.6)		13/45 (28.9)		1/23 (4.4)		6/22 (27.3)		3/23 (13.0)		10/22 (45.5)	
	m(-/+, +/+)	7/53 (13.2)		15/53 (28.3)		2/26 (7.7)		5/27 (18.5)		4/26 (15.4)		11/27 (40.7)	
*UGT1A7*			0.041		0.082		0.049		0.494		0.042		0.868
	*1/*1	1/28 (3.6)		4/28 (14.3)		0/19 (0.0)		1/9 (11.1)		0/19 (0.0)		4/9 (44.4)	
	*1/*2, *2/*2	3/29 (10.3)		8/29 (27.6)		0/13 (0.0)		3/16 (18.8)		2/13 (15.4)		6/16 (37.5)	
	*1/*3, *2/*3, *3/*3	10/41 (24.4)		16/41 (39.0)		3/17 (17.7)		7/24 (29.2)		5/17 (29.4)		11/24 (45.8)	
UGT1A9*22			0.033		0.083		0.22		0.22		0.024		0.843
	w(10/10)	1/30 (3.3)		5/30 (16.7)		0/19 (0.0)		1/11 (9.1)		0/19 (0.0)		5/11 (45.5)	
	m(9/10, 9/9)	13/68 (19.1)		23/68 (33.8)		3/30 (10.0)		10/38 (26.3)		7/30 (23.3)		16/38 (42.1)	

Toxicity grade by the National Cancer Institute Common Toxicity Criteria, version 3.0.

a Ninety-four out of 98 patients could be analyzed for the UGT1A1*27 mutation.

**Table 5.** Diplotypes constructed only with UGT1A1*6, UGT1A7*3, and UGT1A9*22 and their relationships to severe irinotecan toxicity

Diplotypes	Total (%) (n = 98)	GIII-IV diarrhea		GIII-IV neutropenia	
		No. (%)^a^	p-value	No. (%)^b^	p-value
UGT1A1*1/UGT1A1*1	29 (29.6)	1 (7.1)		5 (17.9)	
UGT1A1*1/UGT1A9*22	26 (26.5)	3 (21.4)	0.46	6 (21.4)	0.469
UGT1A1*1/UGT1A1*6 + UGT1A7*3 + UGT1A9*22	20 (20.4)	6 (42.9)	0.024	8 (28.6)	0.205
UGT1A1*1/UGT1A7*3 + UGT1A9*22	6 (6.1)	1 (7.1)	0.614	2 (7.14)	0.553
UGT1A9*22/UGT1A1*6 + UGT1A7*3 + UGT1A9*22	5 (5.1)	2 (14.3)	0.148	2 (7.14)	0.444
UGT1A9*22/UGT1A9*22	2 (2.0)	0	0.733	1 (3.6)	0.492
UGT1A9*22/UGT1A1*6 + UGT1A7*3	2 (2.0)	0	0.733	1 (3.6)	0.492
UGT1A7*3/UGT1A1*6 + UGT1A7*3 + UGT1A9*22	2 (2.0)	0	0.733	0	0.508
UGT1A7*3 + UGT1A9*22/UGT1A1*6 + UGT1A7*3 + UGT1A9*22	2 (2.0)	0	0.857	1 (3.6)	0.492
UGT1A1*6 + UGT1A7*3 + UGT1A9*22 / UGT1A1*6 + UGT1A7*3 + UGT1A9*22	2 (2.0)	1 (7.1)	0.267	2 (7.14)	0.08
UGT1A9*22/UGT1A7*3 + UGT1A9*22	1 (1.0)	0	0.857	0	0.714
UGT1A1*1/UGT1A1*6 + UGT1A7*3	1 (1.0)	0	0.857	0	0.714

a The percentages of grade III‒IV diarrhea were calculated out of the total number of patients who suffered severe toxicities (each out of 14 and 28).

b The percentages of grade III‒IV neutropenia were calculated out of the total number of patients who suffered severe toxicities (each out of 14 and 28).

## Supplementary Materials

Supplementary Table S1. Primers for *UGT1A* PCR

Supplementary Table S2. Haplotype frequency

Supplementary Table S3. Diplotype frequency

Supplementary Table S4. Associations of UGT1A genotypes with treatment response
