# Supplementary material for: The UGT1A9*22 genotype identifies a high-risk group for irinotecan toxicity among gastric cancer patients
Source: Genomics Inform. 2022 Sep 30;20(3):e29. doi: 10.5808/gi.22051 (PMC9576471; doi:10.5808/gi.22051)
Supplement: Supplementary Table S1. — Primers for UGT1A PCR [file gi-22051suppl1.pdf]

Supplementary Table 1. Primers for *UGT1A* PCR

Gene	Polymorphism	rs No.	Primer sequence	
UGT1A1	686C>A (*27)	rs35350960	Forward	TGTCTGGCTGTTCCCACTT
			Reverse	GGGCTAGTTAATCGATCCAAAG
	–53(TA)6>7 (*28)	rs8175347	Forward	TCCCTGCTACCTTTGTGGA
			Reverse	AGGAAAGGGTCCGTCAGC
UGT1A9	–118$(T)_{10}$/$(T)_{9}$ (*22)	rs3832043	Forward	AGGCGAGCCCCAATTTAG
			Reverse	GGCAAAGCCACAGGTCAG
UGT1A7	129N>K (387T>G)	rs17868323	Forward	CACCATTGCGAAGTGCAT
	131R>R/K (391C>A)	rs17863778	Reverse	TTCTTAATGTGCTAAAGGGGAGA
	131R>Q/K (392G>A)	rs17868324		
	208W>R (622T>C)	rs11692021		
UGT1A1	211G>A (*6)	rs4148323		
	–3279T>G (*60)	rs4124874		

PCR, polymerase chain reaction.
