# Supplementary material for: The UGT1A9*22 genotype identifies a high-risk group for irinotecan toxicity among gastric cancer patients
Source: Genomics Inform. 2022 Sep 30;20(3):e29. doi: 10.5808/gi.22051 (PMC9576471; doi:10.5808/gi.22051)
Supplement: Supplementary Table S2. — Haplotype frequency [file gi-22051suppl2.pdf]

Supplementary Table 2. Haplotype frequency

	Haplotype					Frequency (%)	
	*UGT1A9*22* –118$(T)_{10}/(T)_9$	*UGT1A7*	*UGT1A1*60* –3279T/G	*UGT1A1*28* –53(TA)6>7	*UGT1A1*6* 211G/A	Total (n = 382)	Patients who received irinotecan-containing chemotherapy (n = 98)
I	10	TCGT(*1)	T	6	G	48.66	48.98
II	9	GAAC(*3)	T	6	A	17.35	16.84
III	9	GAAT(*2)	G	6	G	15.33	16.84
IV	10	TCGT(*1)	G	7	G	8.66	6.12
V	9	GAAT(*2)	G	7	G	1.98	2.55
VI	9	GAAC(*3)	G	7	G	1.77	4.08
VII	10	GAAC(*3)	T	6	A	1.76	1.53
VIII	9	GAAC(*3)	T	6	G	1.67	0.51
IX	10	TCGT(*1)	G	6	G	0.93	0.51
X	10	TCGT(*1)	T	6	A	0.52	0.00
XI	9	GAAT(*2)	T	6	A	0.39	0.00
XII	10	GAAC(*3)	G	7	G	0.29	1.02
XIII	10	GAAC(*3)	G	6	G	0.26	0.00
XIV	9	GAAT(*2)	T	6	G	0.26	0.51
XV	10	GAAT(*2)	G	6	G	0.17	0.51
