# Supplementary material for: The UGT1A9*22 genotype identifies a high-risk group for irinotecan toxicity among gastric cancer patients
Source: Genomics Inform. 2022 Sep 30;20(3):e29. doi: 10.5808/gi.22051 (PMC9576471; doi:10.5808/gi.22051)
Supplement: Supplementary Table S3. — Diplotype frequency [file gi-22051suppl3.pdf]

Supplementary Table 3. Diplotype frequency

Diplotype	Frequency (%)	
	Total (n = 382)	Patients who received irinotecan-containing chemotherapy (n = 98)
I, I	22.77	24.49
I, II	17.8	17.35
I, III	15.18	16.33
I, IV	8.64	6.12
II, III	5.76	4.08
II, IV	3.66	3.06
I, V	2.36	4.08
I, VIII	2.36	0
III, III	2.09	2.04
III, IV	2.09	1.02
I, VI	1.83	5.1
II, III	1.83	4.08
I, VII	1.57	1.02
III, IX	1.05	1.02

Diplotype frequencies are shown. A total of 41 diplotypes were found, and only those with frequencies over 1% among the 382 patients are shown in the table. The four most common diplotypes were I/I (22.77%), I/II (17.80%), I/III (15.18%), and I/IV (8.64%). The 98 patients who received irinotecan-containing chemotherapy also showed similar results.
