# Supplementary material for: The UGT1A9*22 genotype identifies a high-risk group for irinotecan toxicity among gastric cancer patients
Source: Genomics Inform. 2022 Sep 30;20(3):e29. doi: 10.5808/gi.22051 (PMC9576471; doi:10.5808/gi.22051)
Supplement: Supplementary Table S4. — Associations of UGT1A genotypes with treatment response [file gi-22051suppl4.pdf]

Supplementary Table 4. Associations of *UGT1A* genotypes with treatment response

	Responders[a]					
	Total patients (n = 91)		Weekly regimen (n = 46)		Biweekly regimen (n = 45)	
	No. (%)	p-value	No. (%)	p-value	No. (%)	p-value
*UGT1A1*6*		0.493		0.444		0.35
w (-/-)	9/58 (15.52)		7/32 (21.88)		2/26 (7.70)	
m (-/+, +/+)	7/33 (21.21)		4/14 (28.57)		3/19 (15.79)	
*UGT1A1*27*[b]		0.504		0.575		0.76
w (-/-)	16/85 (18.82)		11/44 (25.00)		5/41 (12.20)	
m (-/+, +/+)	0/2 (0.00)		0/0 (0.00)		0/2 (0.00)	
*UGT1A1*28*		0.582		0.601		0.596
w (6/6)	12/67 (17.91)		8/34 (23.53)		4/33 (12.12)	
m (6/7, 7/7)	4/24 (16.67)		3/12 (25.00)		1/12 (8.33)	
*UGT1A1*60*		0.337		0.245		0.565
w (-/-)	9/44 (20.45)		7/23 (30.43)		2/21 (9.52)	
m (-/+, +/+)	7/47 (14.89)		4/23 (17.39)		3/24 (12.5)	
UGT1A7		0.564		0.54		0.544
*1/*1	3/27 (11.11)		3/19 (15.79)		0/8 (0.00)	
*1/*2, *2/*2	5/21 (23.81)		3/11 (27.27)		2/15 (13.33)	
*1/*3, *2/*3, *3/*3	8/38 (21.05)		5/16 (31.25)		3/22 (13.63)	
*UGT1A9*22*		0.173		0.234		0.266
w (10/10)	3/29 (10.34)		3/19 (15.79)		0/10 (0.00)	
m (9/10, 9/9)	13/62 (20.97)		8/27 (29.63)		5/35 (14.29)	

Out of 91 assessable patients, 16 (17.6%) were considered responders. There were no associations between any *UGT1A* polymorphism and treatment response to irinotecan-containing chemotherapy.

[a] Responder: complete or partial response, according to the guidelines of the Response Evaluation Criteria in Solid Tumors (RECIST) Committee.

[b] Eighty-seven of 91 patients were analyzed for *UGT1A1*27* mutations.
